# Hemifacial Spasm and Blepharospasm: Divergent Social Cognition Performance Despite Similar Neurocognitive Profiles

**DOI:** 10.1002/brb3.70794

**Published:** 2025-08-22

**Authors:** Samet Çelik

**Affiliations:** ^1^ Department of Psychology Bartin University Bartin Türkiye

**Keywords:** behavioral neurology, blepharospasm, hemifacial spasm, neurocognition, social cognition

## Abstract

**Background:**

Hemifacial spasm (HFS) and blepharospasm (BSP) are two neurological disorders characterized by involuntary contractions in the eye and face area. These two diagnostic groups' neurocognitive and social cognition performances are not adequately understood regarding behavioral neurology.

**Objectives:**

This study compared HFS and BSP groups with healthy controls (HCs) regarding neurocognition and social cognition performances.

**Methods:**

Twenty people from each group with equal distribution in terms of age, education level, and gender were included in the study. All participants were administered neuropsychological tests measuring attention, memory, working memory, visual‐spatial skills, and theory of mind skills.

**Results:**

The analyses determined that the neurocognition scores of the HFS and BSP groups were lower than those of the HCs, but there was no significant difference between them. A significant difference was found between the HFS and BSP groups regarding social cognition scores.

**Discussion:**

Consistent with the literature, both clinical groups showed differences in the control group's neurocognitive and social‐cognitive test performances. Unexpectedly, it was found that individuals with HFS and BSP, who progress through different pathophysiological mechanisms, exhibited similar neurocognitive scores, but their social cognition scores diverged. This finding suggests that the two disorders exhibit overlapping impairments in some neural circuits while differing in others. Future studies should focus on social cognition skills to better understand the etiology of these two disorders and distinguish the clinically confounding conditions.

## Graphical abstract

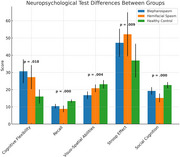

## 1 Introduction

Primary dystonia is the third most common movement disorder (Aita et al. 2022). Although the pathophysiology and pathogenesis of primary dystonias are not fully known, their hyperkinetic movement disorders are characterized by sustained or intermittent muscle contractions characterized by twisting movements or abnormal posture (Albanese et al. 2013; Ferrazzano et al. 2019).

Among the different types of dystonia, blepharospasm (BSP), which is caused by problems with the ocular nerves, stands out (Çakmur 2011). Recent findings in the literature indicate that primary dystonias are more than just movement disorders, as they can also impact and impair cognitive skills as a result of the disorder's progression (Aita et al. 2022; Lange et al. 2017; Steeves et al. 2012). This cognitive impairment occurrence is labeled as non‐motor symptoms (Santangelo et al. 2020; Yang et al. 2016). In dystonia, particularly in BSP, executive impairments, attention deficiencies, and social cognition problems are the subject of extensive research (Stamelou et al. 2012; Bailey et al. 2022; O'Connor et al. 2024). However, findings can be inconsistent; for example, Dias et al. (2009) discovered that those with dystonia had standard cognitive abilities.

Conversely, patients with HFS rarely experience cognitive or emotional challenges, as their condition is mostly related to peripheral nerve damage, with a limited involvement of central neural networks (Setthawatcharawanich et al. 2011). Due to this difference between the two disorders, finding out more about the non‐motor symptoms that occur from different etiologies but have comparable facial hyperkinetic symptoms is essential for their clinical treatment processes and clinical management (Fang et al. 2021). Hemifacial spasm (HFS) and BSP can be present together in certain cases. Also, both disorders are frequently associated with non‐motor symptoms such as low quality of life, depression, anxiety, and problems related to sleep (Dias et al. 2010; Fontenelle et al. 2011; Lawes‐Wickwar et al. 2022). Additionally, a comparison evaluation is driven by the two conditions' similar treatment possibilities.

Several studies conducted in the past 20 years have found that people with HFS and BSP do worse than HCs on tests that measure several cognitive skills (Maggi et al. 2019; Steeves et al. 2012; Yang et al. 2016; Yilmaz and Bilen 2023). There is conflicting evidence regarding the cognitive functions of primary BSP and HFS. Some studies have compared the two groups to gain a better understanding of their causes, progression, and clinical features, and they have found no statistically significant difference (Dias et al. 2009). On the other hand, Lange et al. (2017) found a statistical difference.

Research on the cognitive differences between BSP and healthy control (BSP‐HC) groups and HFS has shown conflicting results in the existing literature review. Additionally, the social‐cognitive capabilities of these populations have not been thoroughly studied. As a result, the goal of this study is to directly compare the neuropsychological test results of persons diagnosed with BSP and HFS to those of an HC group. This approach seeks to address the gaps and discrepancies in the literature, offering a more comprehensive understanding of the cognitive and social‐cognitive profiles associated with these conditions. Based on the findings in the literature, this study addresses the following research questions: (i) Do the cognitive functions of individuals with BSP differ from those of HCs? (ii) Do the cognitive functions of individuals with HFS differ from those of HCs? (iii) Do the cognitive functions of individuals with BSP differ from those with HFS?

## 2 Materials and Methods

### 2.1 Participants

This study included 20 primary BSPs, 20 HFSs, and 20 healthy adults matched with these groups regarding age, education level, and gender. The clinical group was recruited from two different cities and three different hospitals. Individuals diagnosed with BSP‐HFS were diagnosed by neurologists and included in the study in order of their volunteering, considering the inclusion and exclusion criteria.

For individuals diagnosed with BSP‐HFS, (i) comorbidity neurological or psychiatric diagnosis, (ii) the utilization of psychotropic medications or antidepressants deemed to potentially confound the research outcomes, and (iii) who had higher Beck depression and anxiety cut‐off scores, (iv) having a Mini‐Mental State Examination (MMSE) score below 24 were excluded from the study. In addition, botulinum toxin (Btx) treatment had been received 3 months before data collection. HCs were also recruited using the first four exclusion criteria. Finally, the demographic and clinical information of the participants is compared in Table 1.

**TABLE 1:** The comparison of socio‐demographic and clinical characteristics of the sample groups.

Variables		Blepharospasm (*n* = 20)	Hemifacial spasm (*n* = 20)	Healthy control (*n* = 20)	Test statistics	*p*
Age		56.10 ± 10.38	58.30 ± 13.35	51.65 ± 10.98	4.296	0.117^*^
Education level (years)		7.30 ± 3.96	9.40 ± 5.03	8.30 ± 4.12	2.408	0.300^*^
Sex	Male	5	10	9	2.917	0.233^**^
	Female	15	10	11		
Handedness	Left	1	1	1	1.036	0.904^**^
	Right	19	19	18		
	Both	0	1	1		
Duration of disease		12.86 ± 8.61	9.20 ± 7.77		−1.420	0.156^***^
Duration of treatment		9.70 ± 7.90	7.45 ± 6.64		−0.759	0.448^***^
Beck depression score		4.00 ± 2.52	3.47 ± 2.71	4.00 ± 3.02	0.659	0.719^*^
Beck anxiety score		3.67 ± 2.83	2.90 ± 2.16	3.05 ± 2.78	0.575	0.750^*^

* Kruskall–Wallis test, **chi‐square test, and ***Mann–Whitney *U* test, *p* < 0.05.

### 2.2 Clinical Measurements

Demographic and clinical characteristics—including age, sex, educational level, disease duration, Btx treatment duration, and the number of Btx injections—were collected from all patients through face‐to‐face interviews. The Jankovic Rating Scale (JRS) was used for BSP, while the hemifacial spasm severity scale (HSSS) was utilized for HFS. The self‐reported Beck Depression Inventory (BDI) and Beck Anxiety Inventory (BAI) were administered to assess the severity of depression and anxiety symptoms in all participants.

### 2.3 Neuropsychological Assessment

Two certified psychologists administered a standardized neuropsychological test battery to all participants. The tests were administered in accordance with the guidelines and application protocols recommended in the original development and adaptation studies. When determining the administration order, care was taken not to perform tests measuring similar cognitive skills one after the other, thus minimizing the learning effect and interference between tests.

The applications were performed in a quiet clinical environment where external stimuli were controlled. First, the Öktem Verbal Memory Processes Test (Ö‐VMPT) and the WCST were administered, followed by the TMT‐A and B. In the next stage, the Line Orientation Test and the Stroop Test, which assessed executive functions, were administered, followed by the Tower of London Test. Finally, the Reading the Mind in the Eyes Test and the Dokuz Eylül Theory of Mind (9E‐ToM) Index were administered, which measured social cognitive skills. Standard rest breaks were given between the tests, and experienced researchers conducted all applications under similar physical conditions.

This order was specifically designed to both distribute participant fatigue evenly and to ensure that different cognitive domains could be assessed independently. In order to maintain standardization during all test administrations, the instructions and rules specified in the original test manuals were followed completely. In order to ensure consistency between the operators, joint training sessions were organized before the test and the administration's integrity was regularly monitored.

#### 2.3.1 Wisconsin Card Sorting Test (WCST)

In this study, the WCST, finalized by Heaton 1981, is used to evaluate cognitive features such as cognitive flexibility, abstract reasoning, and attention maintenance. The computerized version developed by Çelik et al. (2021, 2023) was used in this research. Once the participant completes the test, the WCST‐computer version gives the researcher 11 subtest scores. These scores are the total number of responses, the total number of errors, the total number of correct responses, the number of categories completed, the total number of perseverative responses, the total number of perseverative errors, the total number of non‐perseverative errors, the percentage of perseverative responses, number of responses used to complete the first category, percentage of conceptual level responses, and failure to maintain set, respectively. The total number of perseverative errors and the number of perseverative responses are sub‐scores used in assessing cognitive flexibility (Miles et al. 2021). An increase in these scores is interpreted as a decrease in the participant's cognitive flexibility. Karzmark (1992) and Perrine (1993) claim that the low percentage of conceptual‐level replies and lack of capacity to sustain set scores reveal insights on working memory and attention. High scores on these subtests indicate that the participant's working memory and attention capacity are lower.

#### 2.3.2 Trial Making Tests

The Trail Making Test is divided into two sections: TMT‐A and TMT‐B, which are designed to examine visual‐motor speed, attention, and executive processes. The TMT‐A measures fundamental visual scanning and psychomotor speed, whereas the TMT‐B evaluates more complicated cognitive functions like set‐shifting and behavioral inhibition (Strauss et al. 2006). In clinical practice, the B/A ratio is often used together with raw scores, and performance on these tests is assessed by the amount of time (in seconds) spent to complete the activity Martin et al. (2003).

The test is a comprehensive instrument that evaluates various domains, including set‐shifting, attention processes, and visual working memory. It is believed that longer completion times are linked to worse cognitive ability (Tombaugh 2004). Because of these features, the TMT is a reliable instrument for neuropsychological research and medical assessments. The amount of time each participant took to complete the exam is measured in seconds, and longer completion time is interpreted as decreased cognitive function.

#### 2.3.3 Stroop Test‐Çapa Version

As stated by Emek‐Savaş et al. (2020), the interference effect, which is the capacity to maintain behavior in the presence of disruptive stimuli, can be measured by the Stroop test. This study utilized the Stroop‐ÇAPA version of the well‐established test. During the procedure, the time it took to complete each activity was measured in seconds, and the participant groups were compared to one another. People who take longer to finish tasks are thought to be less able to handle distractions, which is a sign of poor cognitive ability.

#### 2.3.4 Judgment of Line Orientation (JoLO)

People's visual perception, attention, and spatial orientation are all evaluated on the JoLO test. The maximum possible score on the test is 30, with participants receiving one point for each correct answer. A high JoLO score indicates strong visuospatial ability (Qualls et al. 2000).

#### 2.3.5 Öktem Verbal Memory Processes Test

The Ö‐VMPT is a neuropsychological assessment instrument designed for evaluating an individual's verbal memory capability. The test assesses both short‐term and long‐term memory skills by examining the processes of learning, storing, and recalling verbal information. The test, which consists of 12 distinct score types, was utilized in this study as immediate memory, total learning score, the highest learning score, and delayed recall subscores. As the test is particularly effective in assessing memory processes linked to hippocampal functions, its use in medical contexts is vital, as it contributes to the diagnosis of dementia and other neurocognitive disorders. Additionally, its validity and reliability have been proven by Öktem (2024).

#### 2.3.6 Dokuz Eylül Theory of Mind Index

9E‐ToM is a comprehensive assessment instrument designed to evaluate individuals' mentalization abilities, which refers to the ability to comprehend the thoughts and intentions of others. In total, the test comprises seven stories and three visual drills. In story parts, at first, participants listen to stories presented by others and then respond to questions regarding those stories. At the end of the test, the participants receive a total score ranging from 0 to 18, with a low score signaling poor mentalization capabilities. The 9E‐ToM test is a valid research instrument that objectively and reliably evaluates theory of mind (ToM) capabilities in medical and research contexts (Değirmencioğlu et al. 2018).

#### 2.3.7 The Reading the Mind in the Eyes Test

This test, which is an aspect of the ToM, was developed by Baron Cohen and his associates and evaluates mind reading (Baron‐Cohen et al. 2001). This exam requires participants to analyze and draw conclusions about mental states based on facial features they notice (Bora and Berk 2016).

### 2.4 Statistical Analysis

Continuous variables were stated as mean ± standard deviation, while categorical variables were separated in terms of frequency and percentage. Statistical analysis showed that, according to the results obtained, scores from the neuropsychological test battery were not distributed normally between the participant groups. For this reason, statistical analyses were continued with non‐parametric analyses. We used the Kruskal–Wallis test to compare continuous variables in more than two groups and used Mann–Whitney *U* test results for two groups of comparisons. We used Pearson's or Fisher's exact chi‐square tests to compare categorical variables. A *p*‐value lower than .05 was considered statistically significant for all tests (*p* < 0.05). For multiple comparisons, the false discovery rate (FDR) was performed.

## 3 Results

First of all, we compared the socio‐demographic characteristics of the groups. According to statistics, the three groups were equal in terms of sex, age, and education level. The patients with HFS and patients with BSP are statistically similar regarding clinical features such as disease duration and treatment.

Furthermore, an examination was conducted to determine whether statistical differences in performance on assessments of executive function and social cognition were evident among the three groups. The Kruskal–Wallis test was used to evaluate these differences for each subscore separately. When statistically significant Kruskal–Wallis statistics emerged, subsequent pairwise group comparisons were conducted using the Mann–Whitney *U* test.

The analysis of neurocognitive test performance revealed significant differences among the three groups, specifically regarding learning score, long‐term recall, WCST sub‐scores, JoLO, and Stroop test performances. In pairwise comparisons, it was established that both the BSP and HFS groups exhibited statistically lower performances than the HC group. Conversely, no statistically significant differences in TMT and ToL performances were observed among the groups.

Table 2 presents the mean scores and standard deviations for each subscore across the groups. Notably, except for the JoLO performance, there were no statistically significant executive function scores between the HFS and BSP groups. The JoLO performance of the BSP group was lower than that of the HFS group.

**TABLE 2:** Differences of the neuropsychological profiles between sample groups.

Neuropsychological tests	Subscores	Blepharospasm^A^	Hemifacial spasm^B^	Healthy controls^C^	Test statistics	Group differences	*p*
Ö‐VBPT	Immediate memory	5.21 ± 3.22	4.55 ± 1.50	5.16 ± 1.46	0.990	n.a.	0.610
	Total learning score	99.32 ± 22.48	94.20 ± 28.94	114.58 ± 21.91	8.961	A/C; B/C	0.011
	Hıghest learning score	13.67 ± 2.28	12.10 ± 3.29	13.80 ± 3.80	8.715	A/C; B/C	0.013
	Recall	10.42 ± 2.87	8.80 ± 4.34	13.37 ± 2.17	18.347	A/C; B/C	0.000
WCST	Total number of responses (nTR)	125.21 ± 10.42	126.00 ± 6.96	113.42 ± 18.86	11.750	A/C; B/C	0.003
	Total number of errors (nTE),	66.08 ± 21.38	65.35 ± 23.48	35.21 ± 16.14	18.606	A/C; B/C	0.000
	Total number of correct responses (nTC)	59.14 ± 16.84	60.65 ± 20.66	78.21 ± 7.48	12.930	A/C; B/C	0.002
	Number of categories completed (nCC)	2.00 ± 1.75	2.30 ± 2.03	4.74 ± 1.52	17.873	A/C; B/C	0.000
	Total number of perseverative re‐ sponses (nPR)	36.58 ± 20.59	31.40 ± 19.73	18.42 ± 12.08	7.940	A/C; B/C	0.019
	Total number of perseverative errors (nPE)	30.64 ± 15.86	27.25 ± 15.88	16.00 ± 9.25	8.074	A/C; B/C	0.018
	Total number of nonperseverative errors (nNPE)	34.86 ± 17.81	37.20 ± 19.32	18.63 ± 9.62	11.655	A/C; B/C	0.003
	Percentage of perseverative responses (pPR),	24.46 ± 11.94	21.43 ± 12.25	13.15 ± 6.45	8.366	n.a.	0.140
	Number of responses used to complete the first category (nRC1),	32.20 ± 18.34	23.86 ± 16.55	18.78 ± 10.72	3.934	A/C	0.000
	Percentage of conceptual level responses (pCLR)	33.18 ± 21.63	33.06 ± 22.93	62.92 ± 15.69	18.213	A/C; B/C	0.000
	Failure to maintain set (FMS)	1.286 ± 1.38	1.400 ± 2.16	1.526 ±1.26	1.322	n.a.	0.516
JoLO	Total score	16.75 ± 4.95	20.78 ± 5.12	23.10 ± 5.57	10.873	A/C; A/B	0.004
Stroop test (seconds)		47.22 ± 18.61	52.13 ± 29.18	36.88 ± 22.27	9.424	A/C; B/C	0.009
Trial making tests	A Form	62.59 ± 23.09	69.42 ± 34.78	53.92 ± 26.11	3.198	n.a.	0.202
	B Form	141.04 ± 55.12	154.67 ± 80.16	129.02 ± 74.89	2.199	n.a.	0.333
Reading the mind in the eyes test	Total score	19.23 ± 4.98	15.15 ± 5.80	22.63 ± 4.03	16.898	A/B; A/C; B/C	0.000
9 Eylül theory of mind test	Total score	12.00 ± 1.95	11.10 ± 3.44	13.78 ± 2.87	9.514	A/C; B/C	0.009

Finally, the three groups were compared in terms of social cognition scores. As a result of the statistical analysis, a statistical difference was found between the three groups in terms of social cognition scores (*p* < 0.05). As a result of pairwise comparisons, it was observed that HFS and BSP groups differed in terms of social cognition.

## 4 Discussion

Research findings can be divided into three main categories: (i) patients with HFS and patients with BSP exhibit lower performance than HCs regarding social and neurocognition scores. This main finding is consistent with other studies in the literature: (ii) patients with HFS and patients with BSP do not differ in neurocognition scores; (iii) patients with HFS and patients with BSP differ in terms of their social cognition performance. Accordingly, the social cognition skills of the individual with HFS are lower than those of the BSP.

### 4.1 Do the Cognitive Functions of Individuals with BSP Differ From Those of HCs?

This study demonstrates significant differences between individuals diagnosed with BSP and HCs in memory, cognitive flexibility, visuospatial skills, resistance to distractors, and ToM abilities. These findings suggest that BSP is not merely a movement disorder but also involves widespread cortical and subcortical effects. However, the literature presents conflicting results regarding the nature and prevalence of cognitive impairments in BSP patients.

The results of this study are backed up by the work of Maggi et al. (2019), who noted that patients with BSP perform lower scores on recall tests compared to HCs. Furthermore, a recent meta‐analysis study revealed that individuals with BSP exhibit a reduction in gray matter volume within the parahippocampal gyrus. The observed atrophy in the parahippocampal gyrus functions as a preliminary biomarker for neurodegenerative diseases (Zhang et al. 2022).

Cognitive flexibility is the most extensively studied cognitive domain regarding executive function performance in the literature. Contrary to some previous findings, our data indicate that individuals with BSP exhibit a decreased capacity for cognitive flexibility. Other research works have shown that there are no differences in this area (Alemán et al. 2009; Balas et al. 2006; Maggi et al. 2019). These differences in the dorsolateral prefrontal cortex (DLPFC)‐striatal pathways are linked to poor executive function in BSP and play a part in the disorder's pathophysiology (Shen et al. 2017; Yang et al. 2013; Zhang et al. 2022). For executive networks, especially those that deal with attention processes, the DLPFC is a key hub for connecting them all. Memory and executive function problems have been shown to be caused by less gray matter in the contralateral putamen and thalamus (Etgen et al. 2006; Fabbrini et al. 2008). The findings suggest that BSP is characterized not only by basal ganglia impairment but also by anatomical and functional changes in limbic and cortical structures, which must be considered throughout the progression of the illness (Carbon et al. 2008; Jinnah and DeFazio 2023). Interestingly, no statistically significant differences in this study were observed between the clinical and control groups regarding visual‐motor speed (TMT‐A Form) and the capacities of set‐shifting and behavioral inhibition (TMT‐B Form). It was assumed that individuals with BSP would perform lower than other sample groups due to the presence of motor symptoms. However, when the data in Table 2 is examined, it is seen that individuals with BSP have better performance in both TMT‐A and TMT‐B compared to individuals with HFS; however, there is no statistically significant difference when compared to the HC group. These findings suggest that metacognitive functions such as behavioral inhibition and set‐shifting may be less affected by the mechanisms involved in the pathophysiology of the disease than other cognitive functions. Other possible reasons include The BSP group in our study, which consisted of younger individuals compared to other studies in the literature, and the effects of psychiatric comorbidities, such as depression and anxiety, which are frequently reported in primary dystonia patients, on cognitive functions.

Visuospatial deficits in BSP have been associated with abnormal connectivity patterns in striatal‐frontal and parietal cortices (Battistella et al. 2017; Berardelli et al. 1998). Prell et al. (2013) reported that reductions in gray matter volume in the right inferior occipital gyrus and left fusiform gyrus may contribute to difficulties in facial recognition and visual information processing in dystonia patients (Prell et al. 2013). These brain regions are critical cortical centers for visual information processing (Rossion et al. 2003).

Psychiatric comorbidities, such as depression, emerge as a significant confounding variable in studies of cognitive performance in dystonia patients. Research indicates that depression rates in primary dystonias, including BSP, can reach up to 83% (Zurowski et al. 2013). While some studies suggest mood disorders arise secondarily to dystonia (Ellement et al. 2021), others argue that depression and anxiety often precede dystonia and should be considered primary disorders (Ndukwe et al. 2020). Regardless of this debate, baseline differences in depression scores are frequently observed in studies investigating cognitive impairment in BSP patients (Yang et al. 2021).

Finally, the present study compared social cognition performance between groups. Research on social cognition in BSP is relatively limited. Our study identified significant differences in social cognition performance between BSP and HC groups. However, J. Xu et al. (2023) found no differences in behavioral or neural correlates of facial emotion recognition among BSP patients. Similarly, other studies have reported that individuals with cervical dystonia exhibit lower social cognition performance compared to those with BSP (Rafee et al. 2021; Feuerstein et al. 2023). To our knowledge, this is the first study to report impaired ToM and mentalization abilities in BSP patients. Changes in gray matter volume in the right inferior occipital gyrus and left fusiform gyrus, as reported by Prell et al. (2013), may underlie difficulties in facial expression recognition observed in BSP patients. These brain regions are essential for visual information processing (Rossion et al. 2003). Additionally, Rizzo et al. (2023) highlighted deficits in the medial prefrontal cortex (mPFC) network in hyperkinetic movement disorders, possibly contributing to impaired social cognition skills (Rizzo et al. 2023).

### 4.2 Do the Cognitive Functions of Individuals With HFS Differ From Those of HCs?

Studies examining cognitive impairment in individuals with HFS are significantly fewer compared to those on primary dystonias. This disparity can be attributed to early assumptions that the etiology of HFS was limited to peripheral nervous system dysfunction, particularly involving cranial nerve VII (Bao et al. 2015; Wang and Jankovic 1998). Consequently, early research primarily focused on the secondary psychiatric symptoms associated with HFS.

Current evidence indicates that individuals with HFS exhibit higher rates of depression and anxiety (Kim et al. 2016; Rosenstengel et al. 2012; Tan et al. 2005), obsessive‐compulsive disorder (Fontenelle et al. 2011), and sleep disturbances (Yilmaz and Bilen 2023) compared to the general population. Furthermore, these patients report significantly reduced quality of life (Lawrence et al. 2018). However, recent neuroimaging studies have challenged the notion that the pathophysiology of HFS is solely due to neurovascular conflict (McLaughlin et al. 1999; Miller and Miller 2012). Instead, they highlight structural and functional alterations in central nervous system regions, including anatomical and functional changes in the amygdala and reduced cortical thickness and connectivity dysfunction in the DLPFC and orbitofrontal cortex (OFC) (Bao et al. 2015; Li et al. 2024; Tu et al. 2016; H. Xu et al. 2019).

Based on this evidence, the present study hypothesized that dysfunction in central nervous system structures, beyond peripheral nervous system abnormalities, would contribute to deficits in various cognitive domains, particularly executive functions. Our findings revealed significant reductions in recall, cognitive flexibility, and Stroop performance among individuals with HFS. Contrary to our findings, most studies comparing cognitive domains between HFS patients and HCs report no statistically significant differences in cognitive performance (Yilmaz and Bilen 2023; Yong et al. 2013). However, considering the macrostructural cortical changes observed in HFS, reductions in memory and executive function scores are not surprising. Bao et al. (2015) specifically reported decreased gray matter volume in the parahippocampal gyrus of individuals with HFS. As previously noted, the parahippocampal gyrus is closely associated with episodic memory (Aminoff et al. 2013). Additionally, neuronal loss in key cortical and subcortical regions involved in executive and attentional networks aligns with the observed behavioral deficits (Bao et al. 2015; H. Xu et al. 2019; Li et al. 2024).

Finally, our study found that individuals with HFS differed significantly from HCs in ToM and facial emotion recognition abilities. To our knowledge, no prior studies have reported social cognition deficits secondary to the pathophysiology of HFS. However, emotional problems, including social phobia and interpersonal difficulties due to involuntary facial muscle control, are frequently reported in the HFS population. These issues may contribute to higher rates of depression and anxiety as individuals often experience shame in social interactions and may withdraw from these relationships (Kim et al. 2016; Kim et al. 2019).

The link between the clinical progression of HFS and the observed deficits in social cognition is not yet completely understood. Some researchers claim that deficiencies in social cognition in HFS contribute to depression, whereas others suggest comorbid psychiatric disorders, such as depression and anxiety, which are conditions that result in social isolation and impairments in social cognition. In order to tackle and understand the complex clinical process of HFS more, interaction between cognitive impairments, social cognition deficiency, and the medical process domains should be domains of further research.

### 4.3 Do the Cognitive Functions of Individuals With BSP Differ From Those With HFS?

This study compared patients' neurocognitive and social‐cognitive capacities with BSP and HFS. A limited number of studies in the literature cover and compare the two disorders due to their distinct pathophysiological mechanisms. However, recent literature on neuroimaging reinforces that these facial hyperkinesias share the same structural and anatomical heterogeneities as well (Fang et al. 2021; Chirumamilla et al. 2019). Furthermore, the two disorders share similar features in response to medical therapies (Dong et al. 2019; Lawes‐Wickwar et al. 2022) and psychiatric comorbidities (Kim et al. 2016; Dias et al. 2010). Furthermore, in some cases, BSP and HFS can co‐exist together (Tan et al. 2004). Examining similarities and unique behavioral, cognitive, and neurological aspects of these disorders could provide significant insights into nervous system function and the fundamental causes of these disorders.

According to the limited investigations that were conducted on the two clinical groups, there were no significant differences in neurocognitive features (Setthawatcharawanich et al. 2011; Dias et al. 2009). The present study showed that other than visuospatial skills, there were no major differences between the BSP and HFS groups in memory, set‐shifting, cognitive flexibility, or Stroop performance, which is consistent with the previous findings in the literature. Further, in accordance with Tan et al. (2004) and Fang et al. (2021), the overlapping pathophysiological mechanisms, particularly those that connect BSP and HFS, may be the cause of the common characteristics in neurocognitive profiles across these groups.

Notably, results from the JoLO test indicated that the BSP group demonstrated inferior performance compared to the HFS group. The JoLO test is commonly used to evaluate visuospatial and orientation capacities, and it is quite sensitive to parietal lobe impairments (Treccani et al. 2005). This found difference could be attributable to non‐overlapping mechanisms between the two groups, especially structural and functional deficiencies in the inferior parietal gyrus, left inferior parietal cortex, and left inferior parietal lobule seen in BSP (Etgen et al. 2006; Zhang et al. 2022).

Intriguingly, ToM abilities were lower in the HFS group compared to the BSP group. While the existing literature highlights structural and functional abnormalities, particularly in regions associated with facial emotion recognition processes, such as decreased gray matter volume in the bilateral amygdala and reduced mPFC connectivity in BSP (Fang et al. 2021), our findings indicate poorer ToM performance in the HFS group. This result deviates from our hypothesis and suggests potential differences in the underlying mechanisms driving social cognition deficits in HFS and BSP.

Given the lack of neuroimaging studies directly comparing the social cognitive structures in HFS and BSP, it remains unclear which mechanisms contribute to these observed differences. Additionally, the findings regarding social cognition deficits in these groups warrant replication in future studies. Fang and colleagues (Fang et al. 2021) reported that the HFS group exhibited reduced functional connectivity in the OFC and amygdala compared to the BSP group. These abnormalities may underlie the observed emotional and visuospatial deficits, as the amygdala plays a critical role in integrating efferent and afferent information related to pain, anxiety, fear, reward, and visual processing (Neugebauer et al. 2009; Fang et al. 2021).

## 5 Conclusion

Investigating non‐motor symptoms in movement disorders is important, as they affect patients' daily living activities (Rizzo et al. 2023). However, limited existing research focuses on neurocognition profiles in BSP and HFS groups (Dias et al. 2009; Yilmaz and Bilen 2023). To the best of our knowledge, this study is the first to compare social cognition performances in individuals diagnosed with HFS and BSP.

Studies show that quality of life decreases and depression rates are similar in both clinical groups (Kim et al. 2016). However, it has been reported that emotional problems are especially common in the HFS group, social phobia is common, and involuntary control of facial muscles negatively affects the interpersonal relationships of individuals. Individuals' feeling ashamed of social relationships and being isolated from these relationships may be related to the more frequent occurrence of depression and anxiety (Kim et al. 2019, 2016). However, the relationship between the low performance detected in social cognition features and the course of the pathology and the clinic is not fully understood. While some authors suggest that social cognition deficits in HFS may cause depression, others emphasize that comorbid mental illnesses can lead to isolation and impairment in social cognition.

For this reason, it is thought that focusing on the cognitive skills of individuals diagnosed with BSP and HFS and understanding the diagnosis, treatment, and other pathology‐related comorbid conditions of the patients will guide the clinician in planning the holistic treatment of the patient (Fang et al. 2021; Tan et al. 2004). Particularly considering that these disorders can sometimes be seen in comorbid patients, focusing on the detected social cognition differences may provide an understanding of the etiologies of the disorders.

Our study possesses several methodological strengths. First, our study is the first systematic controlled study examining different cognitive domains. For instance, in some previous studies comparing both clinical groups to HCs, the mean age of participants exceeded 65 years, making it challenging to eliminate the confounding effects of age on cognitive performance (Alemán et al. 2009). In contrast, our study maintained a younger mean age, reducing this potential bias. Furthermore, mood disorders, which are known to impact cognitive performance and are frequently comorbid with both HFS and BSP, were entirely excluded from our sample.

Similarly, the potential effects of Btx injections—particularly on mood and cognition—were controlled by ensuring that the neuropsychological assessments were conducted consistently following treatment for all participants in both clinical groups. Most importantly, while previous studies comparing BSP and HFS to HC or each other have focused on specific cognitive domains (Ferrazzano et al. 2019) and largely overlooked social cognition, our research comprehensively examined a wide range of cognitive domains, including social cognitive abilities.

Despite these strengths, the primary limitation of our study is the relatively small sample size. In our study, the participants were not grouped using the matched‐subjects design method, where demographic characteristics are precisely matched. As seen in Table 1, although there was no statistically significant difference between the groups in terms of age, the fact that a relatively small sample group was used may have affected the findings more than studies with large samples. The stringent inclusion and exclusion criteria designed to ensure a homogeneous sample resulted in fewer eligible participants.

Finally, HFS and BSP are the most common movement disorders of the craniofacial region, and the relationship between motor and non‐motor symptoms in these diseases is still being investigated. Based on the reports from neurodegenerative diseases, future studies must examine the relationship between motor and non‐motor symptoms, especially in HFS and BSP, to elucidate their importance in the diagnostic process and develop treatment strategies. Such studies will contribute significantly to a better understanding of the disease's pathophysiology and improve patients' quality of life.

## Author Contributions

**Samet Çelik**: conceptualization, investigation, funding acquisition, writing–original draft, methodology, formal analysis, writing–review and editing.

## Ethics Statement

Ethical approval was obtained from the Bartin University Social and Humanities Ethics Committee (Approval number: 2022‐SBB‐0445). We confirm that we have read the journal's position on issues involved in ethical publication and affirm that this work is consistent with those guidelines.

## Conflicts of Interest

The author declares no conflicts of interest.

## Consent

Written informed consent was obtained from all participants before starting data collection.

## Peer Review

The peer review history for this article is available at https://publons.com/publon/10.1002/brb3.70794.

## Data Availability

If requested, the authors will provide the raw data.
